# Fungal immunomodulatory protein FIP-fve mitigates airway inflammation and metabolic dysfunction in an obese allergic asthma model

**DOI:** 10.1038/s41598-025-18166-9

**Published:** 2025-09-25

**Authors:** Chang-Hung Hsiao, Jiunn-Liang Ko, Ting-Shuan Wu, Ko-Huang Lue, Chia-Ta Wu

**Affiliations:** 1 Department of Pediatric, Cheng Ching Hospital, Taichung, Taiwan; 2 Institute of Medicine, Chung Shan Medical University, Taichung, Taiwan; 3 Department of Biomedical Sciences, Chung Shan Medical University, Taichung, Taiwan; 4 Department of Medical Research, Chung Shan Medical University Hospital, Taichung, Taiwan; 5 Division of Allergy, Asthma and Rheumatology, Department of Pediatrics, Institute of Allergy, Immunology, and Rheumatology, Chung Shan Medical University Hospital, Taichung, Taiwan; 6 School of Medicine, Chung Shan Medical University, Taichung, Taiwan; 7 Department of Emergency Medicine, Changhua Christian Hospital, Changhua, Taiwan

**Keywords:** Obesity, Asthma, High-fat diet, FIP-fve, Metabolic dysregulation, Immunology, Medical research

## Abstract

Obesity is a growing global health concern that exacerbates the risk and severity of allergic asthma, yet effective therapies and mechanistic understanding remain limited. This study investigates the therapeutic potential of Flammulina velutipes immunomodulatory protein (FIP-fve) in a murine model of obesity-aggravated allergic asthma. BALB/c mice were fed a high-fat diet (HFD) and sensitized with Dermatophagoides pteronyssinus (Der p) to induce obesity-associated allergic airway inflammation. FIP-fve was administered orally during the sensitization phase. Body weight, serum metabolic parameters (cholesterol, glucose, ALT), airway hyper-responsiveness (AHR), serum cytokine profiles, and lung histopathology were evaluated. FIP-fve treatment significantly reduced HFD-induced body weight gain and normalized serum cholesterol, glucose, and ALT levels. In the obese asthma model, FIP-fve markedly attenuated Der p-induced AHR and decreased serum levels of pro-inflammatory and allergic markers, including IL-6, IL-33, osteopontin, and VCAM-1. Cytokine array analysis revealed that FIP-fve reversed the upregulation of multiple inflammatory, metabolic, and angiogenic cytokines induced by HFD and allergen exposure. Histological analysis confirmed reduced inflammatory cell infiltration and tissue remodeling in the lungs of FIP-fve-treated mice. FIP-fve exhibits multi-targeted protective effects against obesity-aggravated allergic asthma by modulating systemic metabolism, suppressing airway inflammation, and regulating key cytokine networks. These findings suggest that FIP-fve holds promise as a novel therapeutic candidate for the management of obesity-related asthma and its associated metabolic dysfunction.

## Introduction

With rapid advancements in technology and improvements in quality of life, dietary habits have shifted toward increased consumption of high-fat, highly processed foods, contributing to a global rise in overweight and obesity. Recent studies further indicate that over 1.5 billion adults are overweight, with at least 500 million classified as obese^1^. The prevalence of childhood obesity-related diseases is also rising in both developed and developing countries, making childhood obesity a major public health crisis^2,3^. By 2025, it is estimated that approximately 167 million individuals will experience health complications related to overweight or obesity. In Taiwan, the overweight and obesity rate among adults aged 18 and above has risen from 38% in 2009 to 50.3% in 2020, reaching a record high^4^. This means that nearly one in two adults in Taiwan is either overweight or obese, highlighting the severity of the obesity issue.

Obesity can lead to chronic inflammation, significantly impacting various bodily functions, including respiratory health. Many studies suggest that obesity is a critical risk factor for numerous chronic diseases, particularly type 2 diabetes, cardiovascular diseases, and even certain types of cancer^1,2^. Additionally, large-scale epidemiological studies have found that overweight individuals have a higher prevalence of asthma compared to those with normal weight. The secretion of leptin, a hormone associated with obesity, and leptin has been shown to promote airway inflammation and is associated with increased asthma risk and severity. Clinical and experimental studies indicate that higher leptin levels are linked to greater airway hyperresponsiveness and more severe asthma phenotypes, potentially through pro-inflammatory signaling and immune modulation^5^. Obesity is one of risk factor for asthma in adults, with epidemiological data showing a higher prevalence and increased severity of asthma among obese individuals. Recent studies demonstrate a clear dose-response relationship between BMI and incident asthma in adults and highlight the role of adipokines such as leptin in mediating airway inflammation and asthma risk^6–9^. Furthermore, some studies suggest that obesity contributes to the development of allergic rhinitis^10^. These findings collectively imply that obesity is a significant factor in the worsening or poor control of respiratory allergic diseases. Underscoring the clinical importance of addressing obesity in asthma management.

Fungal immunomodulatory protein from Flammulina velutipes (FIP-fve)^11,12^ is a purified protein extracted from the edible mushroom Flammulina velutipes. It has been reported to exhibit immunomodulatory, anti-inflammatory, and anti-tumor properties. Our previous studies^13–15^ have confirmed that FIP-fve effectively alleviates allergic airway inflammation caused by allergens. However, its efficacy in cases where both obesity and allergic inflammation coexist remains unexplored. Therefore, this study aims to investigate this aspect.

Previous research on the relationship between obesity and respiratory allergic diseases, including asthma^2–10^, has primarily relied on big data analysis or observational studies. This study aims to evaluate whether FIP-fve can ameliorate airway inflammation and metabolic dysfunction in a mouse model of obesity-induced allergic asthma. We employed a combined high-fat diet and dust mite (Der p) sensitization model to more comprehensively examine the interplay between obesity and allergic responses. Notably, this work represents the first attempt to evaluate the therapeutic potential of FIP-fve under conditions where metabolic dysregulation contributes to allergic airway inflammation.

## Results

### Body weight changes in each group of mice and the effects of FIP-fve on body weight

**Fig. 1:**
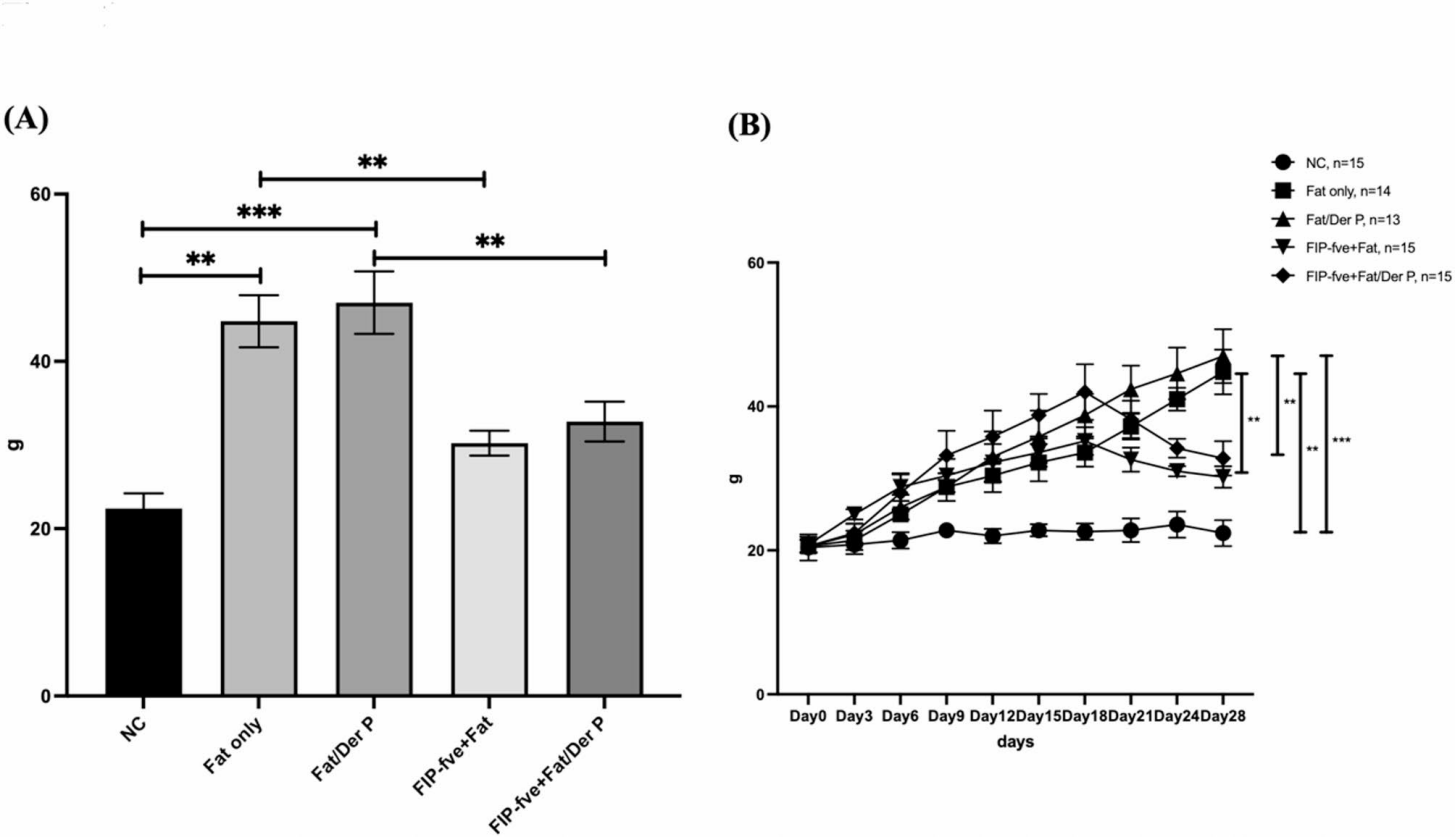
Body Weight Changes in Each Group of Mice and the Effects of FIP-fve on Body Weight **1A**: shows the body weight of mice on the day of sacrifice. The results indicate that the groups receiving a high-fat diet alone or a high-fat diet combined with allergen sensitization exhibited a significant increase in body weight compared to all other groups. Meanwhile, the two groups treated with FIP-fve showed a notable reduction in body weight, bringing their weight closer to that of the NC group. **1B**: illustrates the time-dependent body weight changes in each group. Body weight measurements were recorded every three days throughout the experimental period. As shown in Figure B, after the administration of FIP-fve began on day 14 of the experiment, a decreasing trend in body weight was observed from day 21 onward. NC: Normal Control, Fat only: High-fat diet group, FIP-fve + Fat: FIP-fve-treated high-fat diet group, Fat/Der P: High-fat diet with Der p sensitization group, FIP-fve + Fat/Der P: FIP-fve-treated, high-fat diet with Der p sensitization group. Data are mean ± SD; *P < 0.05, **P < 0.01, ***P < 0.001 indicate significant differences between groups as shown.

Mice fed a high-fat diet alone or combined with allergen sensitization exhibited significant weight gain compared to control groups. Notably, FIP-fve treatment significantly mitigated this weight gain, effectively bringing body weights closer to normal control (NC) levels. Moreover, longitudinal analysis showed that body weight reduction became apparent from day 21 onward, following FIP-fve administration initiated on day 14. These results suggest a potent anti-obesity effect of FIP-fve in the context of allergic asthma induced by obesity (Fig. 1).

### Serum biochemical profiles of each group of mice

**Fig. 2:**
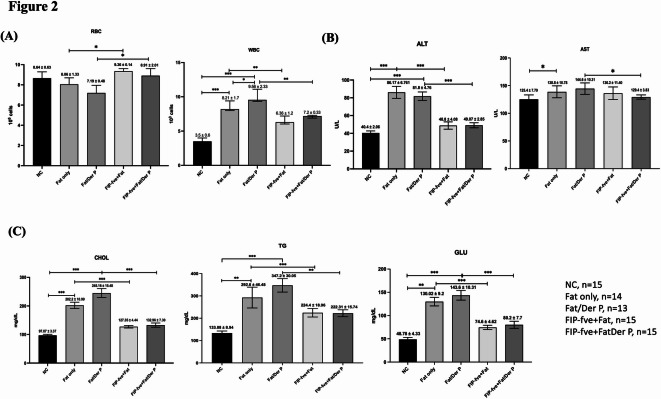
Serum Biochemical Profiles of Each Group of Mice. (**A**) Red blood cell (RBC) and white blood cell (WBC) counts. The Fat only and Fat/Der P groups showed reduced RBC and elevated WBC counts compared to normal controls (NC). FIP-fve treatment partially restored RBC levels and significantly decreased WBC counts in both the FIP-fve + Fat and FIP-fve + Fat/Der P groups. (**B**) Serum alanine aminotransferase (ALT) and aspartate aminotransferase (AST) activities. ALT was significantly increased in the Fat only and Fat/Der P groups but reduced after FIP-fve intervention. AST levels were moderately increased with high-fat diet and allergen exposure but showed no significant changes after FIP-fve treatment. (**C**) Serum cholesterol (CHOL), triglycerides (TG), and glucose (GLU). The Fat/Der P group displayed markedly higher CHOL, TG, and GLU compared to NC. FIP-fve treatment significantly lowered these parameters, approaching levels seen in NC mice. Data are mean ± SD; *P < 0.05, **P < 0.01, ***P < 0.001 indicate significant differences between groups as shown. NC: normal Control; Fat only: high-fat diet group; Fat/Der P: high-fat diet with Der p sensitization; FIP-fve + Fat: FIP-fve-treated high-fat diet group; FIP-fve + Fat/Der P: FIP-fve-treated, high-fat diet with Der p sensitization group. Sample sizes for each group are indicated in the figure.

Figure 2 demonstrates several important trends regarding blood cell counts and metabolic parameters in the study groups. The red blood cell (RBC) count was decreased in the group receiving a high-fat diet combined with Der p sensitization (Fat/Der p) compared to the normal control group, suggesting that the combination of metabolic and allergic stress may impair erythropoiesis or red cell stability. In contrast, white blood cell (WBC) counts were elevated in both the high-fat diet alone (Fat only) and the Fat/Der p groups, indicating an increase in systemic inflammation as a result of dietary and allergic challenges. Importantly, treatment with FIP-fve (FIP + Fat and FIP + Fat/Der p) led to a significant reduction in WBC counts, bringing them closer to the levels found in the control group, and highlighting the anti-inflammatory effect of FIP-fve. Metabolic assessments revealed that both cholesterol and blood glucose levels were significantly increased under conditions of combined high-fat diet and allergen sensitization. However, administration of FIP-fve effectively normalized these metabolic parameters, with values that closely matched those observed in the normal controls. This finding underscores the potential of FIP-fve to correct the metabolic disturbances caused by obesity and allergic exposure. Regarding liver function, the ALT level was highest in the high-fat diet alone group, indicating possible mild liver stress induced by high-fat feeding. This elevation in ALT was alleviated upon FIP-fve treatment, suggesting a protective effect on hepatic function.

### Airway hyper-responsiveness (AHR), der p-specific ige, and inflammatory cell infiltration in each group

**Fig. 3:**
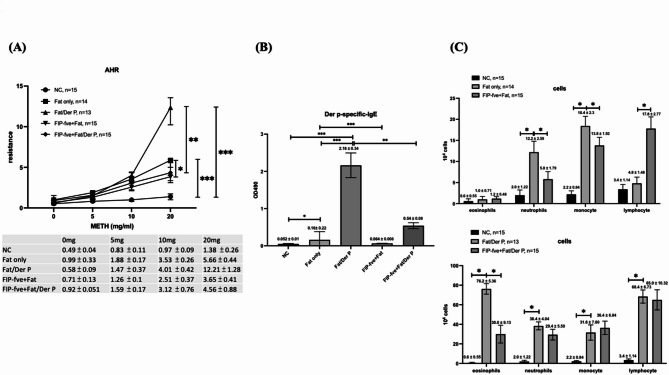
Airway Hyper-Responsiveness (AHR), Der p-specific IgE, and Inflammatory Cell Infiltration in Each Group. (**A**) Airway resistance in response to methacholine (METH) increased in Fat only and Fat/Der P groups, with the highest response in Fat/Der P. FIP-fve intervention significantly reduced resistance in both FIP-fve + Fat and FIP-fve + Fat/Der P groups. Resistance values (mean ± SD) at each METH dose are shown in the accompanying table. (**B**) Serum Der p-specific IgE was markedly elevated in Fat/Der P mice and significantly reduced by FIP-fve treatment. (**C**) Inflammatory cell counts in bronchoalveolar lavage fluid (BALF): In Fat only and Fat/Der P groups, neutrophil, monocyte, lymphocyte, and eosinophil numbers were increased; FIP-fve treatment reduced these inflammatory cell populations in both high-fat and high-fat plus allergen conditions. Data are mean ± SD. Statistical significance: *P < 0.05, **P < 0.01, ***P < 0.001. Sample sizes are indicated in the figure legend.

Figure 3 A shows that the NC group exhibited the lowest and most stable airway resistance across all methacholine concentrations, confirming normal airway reactivity. The Fat only group displayed a moderate increase in airway hyper-responsiveness, with resistance values rising especially at higher methacholine doses (10 and 20 mg/mL), indicating that a high-fat diet alone can induce mild airway dysfunction. The Fat/Der p group had the highest airway resistance at all methacholine concentrations, demonstrating that combined high-fat diet and Der p allergen exposure substantially enhance airway hyper-responsiveness. Treatment with FIP-fve (both FIP-fve + Fat and FIP-fve + Fat/Der p groups) resulted in significantly lower airway resistance compared to their untreated counterparts, although values were still elevated relative to NC. This suggests that FIP-fve supplementation effectively ameliorates, but does not completely normalize, airway hyper-responsiveness caused by metabolic and allergic triggers.

As shown in Fig. 3B, Der p-specific IgE levels were markedly elevated only in the Fat/Der p group, confirming successful sensitization and pronounced allergic response. Both FIP-fve + Fat and FIP-fve + Fat/Der p groups showed suppressed Der p-specific IgE levels compared to Fat/Der p, indicating the protective effect of FIP-fve against allergen-induced IgE production.

Figures 3 C (upper and lower panels) reveal inflammatory cell profiles in the bronchoalveolar lavage fluid (BALF). In both the Fat only and Fat/Der p groups, eosinophil, neutrophil, and monocyte counts were significantly increased compared to NC, and these changes were most pronounced in Fat/Der p, consistent with severe allergic airway inflammation. Notably, the FIP-fve + Fat/Der p group exhibited significant reductions in the numbers of eosinophils, neutrophils, and monocytes, as well as a partial normalization of lymphocyte populations, compared to Fat/Der p. This indicates that FIP-fve not only reduces airway hyper-responsiveness but also effectively mitigates inflammatory cell infiltration associated with both obesity and allergic asthma.

In allergy-related cytokines (Table 1), Fat only led to significant increases in multiple BALF cytokines, notably IL-4, IL-5, IL-6, IL-12, IL-13, IL-17, IL-33, TNF-α, and IFN-γ, compared to the normal chow group (NC) (all *P* < 0.05 or *P* < 0.01). The combination of high-fat diet and Der p sensitization (Fat/Der P) resulted in a marked further elevation of Th2-type cytokines (IL-4, IL-5, IL-13, IL-33), as well as IL-6, IL-17, and TNF-α, compared to Fat only (all *P* < 0.001), reflecting the synergistic enhancement of airway inflammation by metabolic and allergic stressors.

**Table 1:** Expression of allergy-related cytokines in BALF in each group.

	NC	Fat only	FIP-fve + Fat	Fat/Der *P*	FIP-fve + Fat/Der *P*
IL4	13.2 ± 3.27 ***/+++	27.2 ± 4.02	22.8 ± 3.70	67.2 ± 4.76 ***	32.8 ± 2.95 +++
IL5	9.8 ± 2.95 **/+++	23.4 ± 9.21	23.4 ± 9.21	342.4 ± 32.35 ***	110.8 ± 1.06 +++
IL6	15.4 ± 3.21 **/+++	33.0 ± 13.06	33.0 ± 13.06	327.6 ± 26.51 ***	99.8 ± 8.07 +++
IL12	9.4 ± 1.34 ***/+++	32.8 ± 6.06	28.6 ± 5.32	37.4 ± 8.26	34.2 ± 3.56
IL13	3.2 ± 1.48 ***/+++	29.6 ± 5.81	29.6 ± 5.81	89.2 ± 11.26 ***	44.8 ± 4.27 +++
IL17	3.6 ± 1.14 ***/++	39 ± 4.95	20.0 ± 1.58 **	129.8 ± 15.51 ***	60.2 ± 3.83 +++
IL33	3.2 ± 1.1 ***/+++	24.0 ± 4.06	19.8 ± 1.79	205.8 ± 16.33 ***	126.4 ± 12.72 +++
TNF-a	3.4 ± 1.14 ***/+++	44.8 ± 4.44	28.6 ± 3.51 **	172.2 ± 25.78 ***	99.2 ± 8.26 +++
IFN-r	10.8 ± 1.79 **/+	17.4 ± 2.70	23.8 ± 4.38 *	16.4 ± 1.82	56.6 ± 4.34 +++

Data are presented as mean ± standard deviation (pg/mL).

NC: Normal chow control, Fat only: High-fat diet group, FIP-fve + Fat: FIP-fve-treated high-fat diet group, Fat/Der P: High-fat diet with Der p sensitization group, FIP-fve + Fat/Der P: FIP-fve-treated, high-fat diet with Der p sensitization group, BALF: Bronchoalveolar lavage fluid.

***P < 0.001, **P < 0.01, *P < 0.05 vs. Fat only group; +++*P* < 0.001, ++*P* < 0.01, +*P* < 0.05 vs. Fat/Der P group.

Treatment with FIP-fve substantially reduced the levels of most measured cytokines under both conditions. In FIP-fve + Fat, several cytokines showed significant decreases versus Fat only (e.g., TNF-α, IL-17 A, IFN-γ; *P* < 0.01 or *P* < 0.05). In FIP-fve + Fat/Der P, all tested cytokines were significantly lower than in the Fat/Der P group (all *P* < 0.001), including IL-4, IL-5, IL-6, IL-13, IL-17, IL-33, TNF-α, and IFN-γ. These results demonstrate that FIP-fve effectively alleviates the exacerbated airway inflammatory cytokine response induced by the combination of obesity and allergic stimulus in this murine model.

### Cytokine array analysis in each group of mice

#### A. The difference in normal, fat diet and fat diet plus der p group

**Fig. 4:**
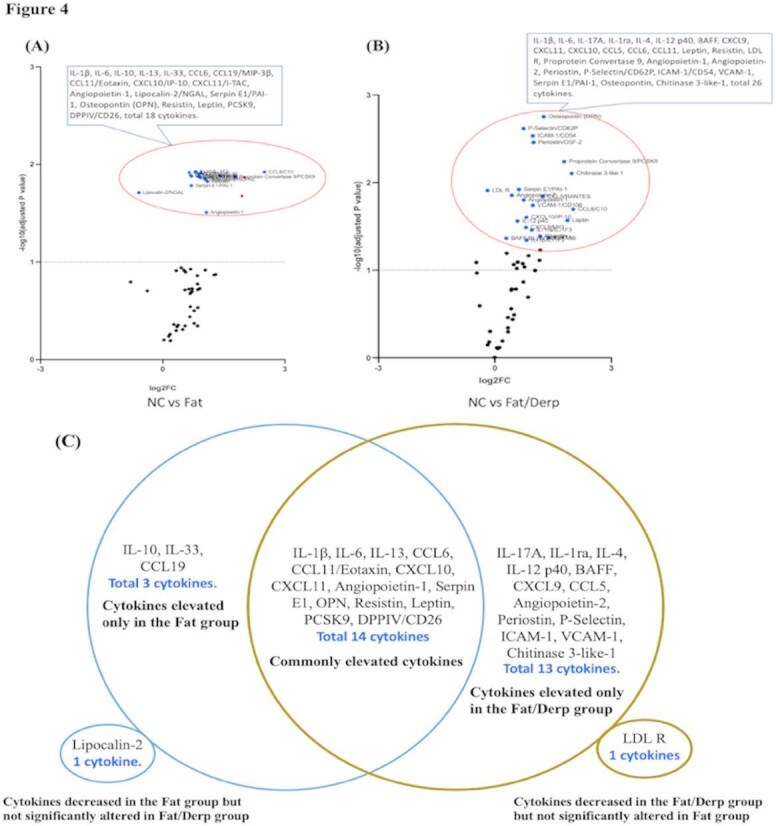
The difference in Normal, Fat diet and Fat diet plus Der p group. Volcano plots and Venn diagram illustrating differential cytokine regulation induced by a high-fat diet alone or combined with allergen sensitization. (**A**-**B**) Volcano plots depict serum cytokine profiles comparing (**A**) normal control (NC) vs. high-fat diet (Fat), and (**B**) NC vs. high-fat diet combined with Der p sensitization (Fat/Derp). Significant cytokines (blue dots) are identified by fold-change and adjusted p-value. (**C**) The Venn diagram summarizes these findings, highlighting cytokines uniquely or commonly altered in each experimental condition. Notably, allergen sensitization amplifies cytokine responses, including inflammation, immune regulation, and metabolic dysfunction.

In the result, we first performed volcano plot analyses to compare cytokine expression among Normal, Fat diet, and Fat/Derp groups, identifying cytokines with statistically significant alterations. Results clearly indicated distinct cytokine expression profiles between the Fat and Fat/Derp groups (Fig. 4).

In the Fat-only group (Fig. 4A), high-fat diet significantly induced multiple cytokines associated with inflammatory responses and immune modulation (IL-1β, IL-6, IL-10, IL-13, IL-33), chemokines and immune cell migration (CCL6, CCL19, CCL11, CXCL10, CXCL11), angiogenesis and vascular function (Angiopoietin-1, Lipocalin-2/NGAL), tissue remodeling and repair (Serpin E1/PAI-1, Osteopontin/OPN), and metabolism and adiposity (Resistin, Leptin, PCSK9, DPPIV/CD26). In total, 18 cytokines exhibited significant changes compared to the Normal diet group, with all cytokines elevated except Lipocalin-2/NGAL, which showed a significant reduction.

Furthermore, in the high-fat diet combined with allergen sensitization (Fat/Derp group) (Fig. 4B), 26 cytokines demonstrated significant alterations compared to the Normal group. These cytokines include those related to inflammatory responses and immune modulation (IL-1β, IL-6, IL-17 A, IL-1ra, IL-4, IL-12 p40, BAFF), chemokines and immune cell migration (CXCL9, CXCL11, CXCL10, CCL5, CCL6, CCL11), metabolism, endocrine regulation, and adiposity (Leptin, Resistin, LDL R, Proprotein Convertase 9), angiogenesis and vascular function (Angiopoietin-1, Angiopoietin-2, Periostin, P-Selectin/CD62P, ICAM-1/CD54, VCAM-1), and tissue remodeling and repair (Serpin E1/PAI-1, Osteopontin, Chitinase 3-like-1).

In Fig. 4C clearly indicates substantial differences in cytokine regulation between a high-fat diet alone and a high-fat diet combined with allergen sensitization. The Fat/Derp group showed more extensive and complex cytokine changes compared to the Fat group alone, particularly involving immune regulation, vascular function, and tissue remodeling factors. These results suggest that allergen sensitization further intensifies and expands the immuno-metabolic dysregulation induced by a high-fat diet.

#### B. The effect of FIP-fve in fat diet group

**Fig. 5:**
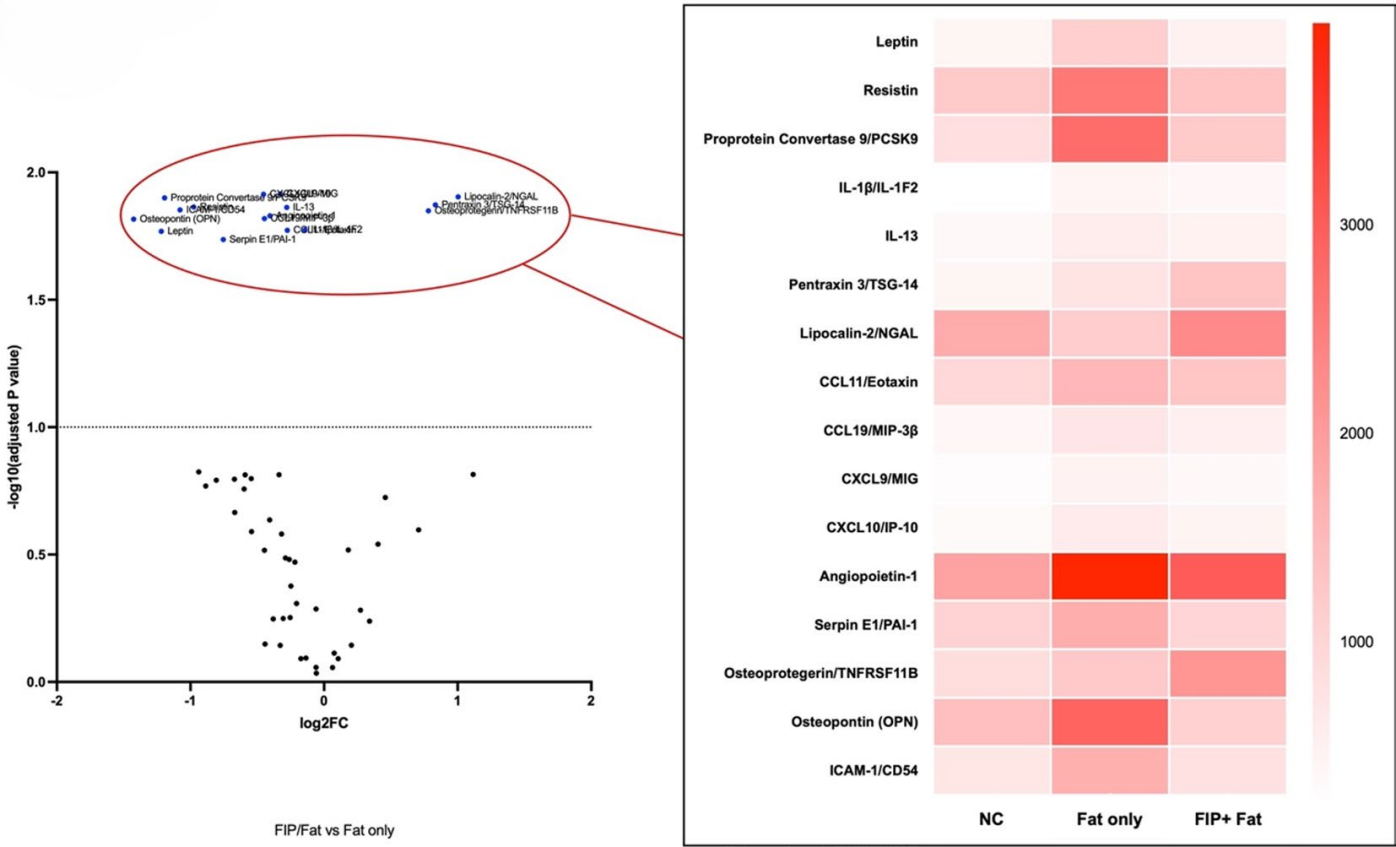
The effect of FIP-fve in Fat diet group. The heatmap and volcano plot illustrate the changes in multiple metabolic and inflammatory markers among groups. High-fat diet (Fat only) increased the expression of several proteins, including leptin, resistin, and PCSK9, compared to controls. FIP-fve treatment (FIP + Fat) reduced most of these elevated markers, notably leptin, resistin, and PCSK9. Some proteins, such as pentraxin-3, lipocalin-2, and osteoprotegerin, were higher in the FIP + Fat group, suggesting FIP-fve may have selective, context-dependent effects.

In the result (Fig. 5; Table 2), volcano plot and heatmap analyses were used. Compared to the NC (normal chow) group, the Fat only group exhibited marked increases in multiple pro-inflammatory and metabolic markers in BALF, including leptin, resistin, proprotein convertase 9 (PCSK9), pentraxin-3 (TSG-14), CCL11/eotaxin, CCL19/MIP-3β, CXCL9/MIG, CXCL10/IP-10, angiopoietin-1, serpin E1/PAI-1, osteoprotegerin (TNFRSF11B), osteopontin (OPN), and ICAM-1/CD54. Notably, angiopoietin-1, leptin, resistin, and PCSK9 showed the most prominent upregulation in the Fat only group, as visualized by the intense colorimetric signal on the heatmap.

**Table 2:** Fold change during fat only and FIP-fve + fat groups.

	Fat-Mean	FIP-fve + FAT-Mean	log2FC-F + FAT	*P*-value
Leptin	1080.95 ± 133.6	464.65 ± 52.4	−1.218083479	0.002
Resistin	2564.7 ± 98.5	1304 ± 102.3	−0.97584621	0.006
Proprotein Convertase 9	2750.2 ± 32.1	1201.9 ± 49.6	−1.194219672	0.009
IL-1β/IL-1F2	422.7 ± 46.1	382.55 ± 10.3	−0.143985789	0.010
IL-13	593.2 ± 58.8	488.7 ± 61.5	−0.279569493	0.011
Pentraxin 3/TSG-14	734.05 ± 64.3	1308.25 ± 79.66	0.833688018	0.020
Lipocalin-2/NGAL	1146.25 ± 97.6	2297.55 ± 326.54	1.003174524	0.021
CCL11/Eotaxin	1540.7 ± 64.8	1273 ± 72.2	−0.275353553	0.022
CCL19/MIP-3β	715.9 ± 13.7	525.6 ± 68.44	−0.445792804	0.024
CXCL9/MIG	457.6 ± 45.2	365.15 ± 15.9	−0.325597822	0.025
CXCL10/IP-10	597.05 ± 55.98	436.25 ± 57.88	−0.452696624	0.025
Angiopoietin-1	3966.95 ± 261.98	2997.1 ± 202.83	−0.404462993	0.026
Serpin E1/PAI-1	1698.35 ± 54.8	1006.7 ± 78.87	−0.754499984	0.032
Osteoprotegerin/TNFRSF11B	1224.2 ± 171.5	2103.95 ± 368.49	0.781261146	0.036
Osteopontin (OPN)	2887.65 ± 99.42	1075.25 ± 25.6	−1.425223758	0.038

All values are shown as mean ± standard deviation (SD).

FAT: High-fat diet (Fat-only group). FIP-fve + FAT: FIP-fve-treated high-fat diet group.

Statistical significance: *P* < 0.05 is considered significant.

Following FIP-fve treatment (FIP + Fat group), several of these upregulated markers were clearly reduced. Particularly, leptin, resistin, and PCSK9 demonstrated obvious decreases compared to the Fat only group, indicating FIP-fve’s ability to ameliorate obesity-associated metabolic and inflammatory alterations. However, some proteins, such as pentraxin-3 (TSG-14), lipocalin-2 (NGAL), and osteoprotegerin (OPG), appeared relatively increased in the FIP + Fat group, suggesting context-dependent regulatory effects.

#### C. The effect of FIP-fve in fat diet plus der p group

**Fig. 6:**
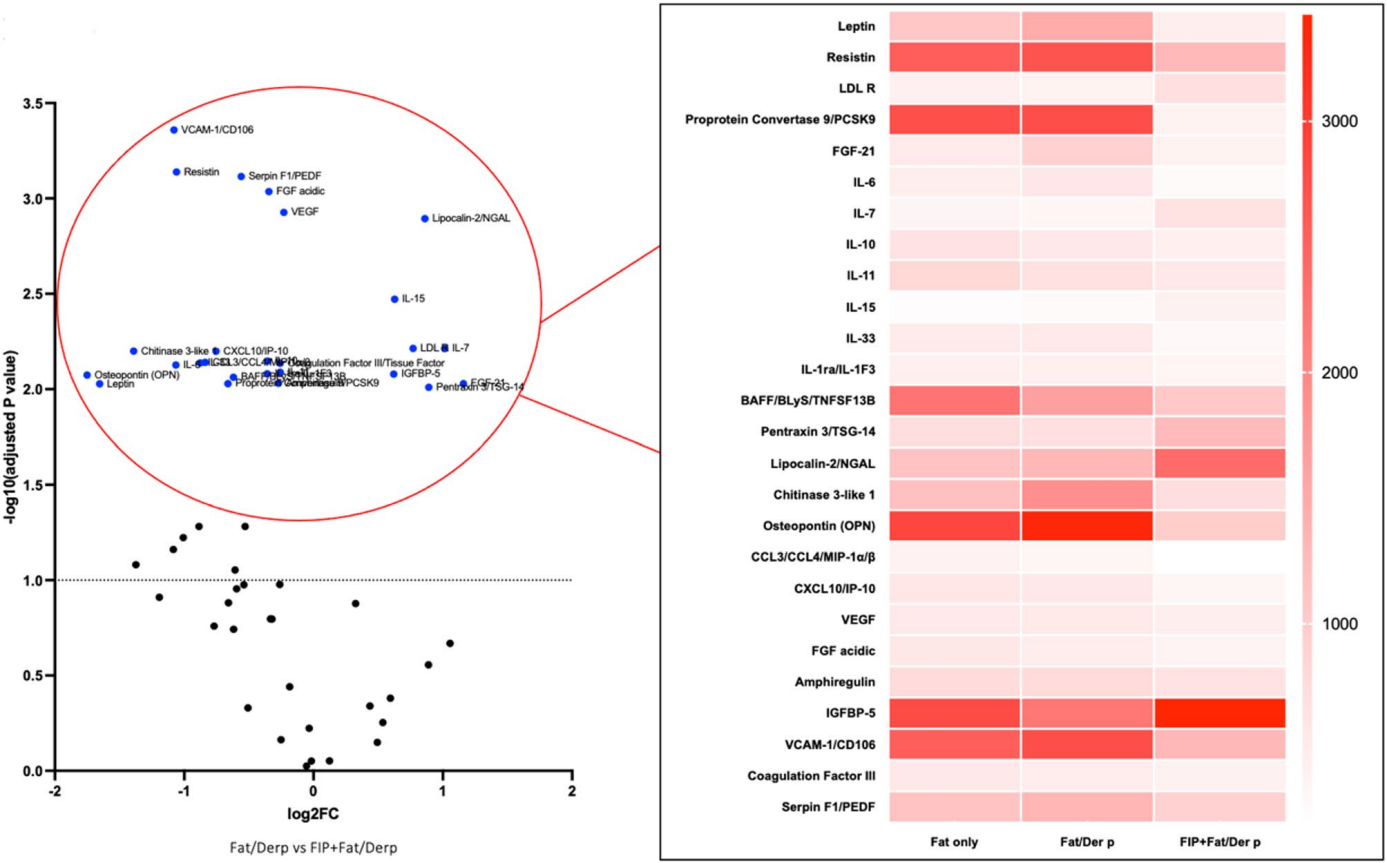
The effect of FIP-fve in Fat diet plus Der p group. Volcano plot and heatmap analyses illustrate the impact of FIP-fve on cytokine expression in bronchoalveolar lavage fluid (BALF). Compared to the Fat/Der P group, FIP-fve treatment markedly reduced multiple inflammatory, immune, vascular, and metabolic markers—including VCAM-1, resistin, IL-6, leptin, IL-33, BAFF, chitinase 3-like-1, and osteopontin. Some factors such as LDLR, IGFBP-5, and lipocalin-2 were higher after FIP-fve intervention, possibly indicating roles in tissue repair.

In the result (Fig. 6; Table 3), FIP-fve treatment in mice exposed to both a high-fat diet and Der p significantly reduced many cytokines associated with inflammation, immune regulation, vascular function, and tissue remodeling—such as VCAM-1, resistin and VEGF—compared to the untreated group. The heatmap further confirms marked decreases in key inflammatory and metabolic markers including leptin, resistin, PCSK9, IL-6, BAFF, chitinase 3-like-1, and osteopontin (OPN) after FIP-fve intervention. Notably, some factors (e.g., IGFBP-5 and LDLR) were increased, suggesting FIP-fve might also promote tissue repair or protective mechanisms.

**Table 3:** Fold change during fat/der P and FIP-fve + fat/derP groups.

	Fat/Der *P*-Mean	FIP-fve + Fat/DerP-Mean	log2FC-F + FAT/Derp	*P*-value
Leptin	1496.8 ± 26.23	475.65 ± 21.35	−1.65390918	0.038
Resistin	2688.8 ± 65.64	1290.45 ± 64.68	−1.059088204	0.007
LDL R	404.15 ± 58.1	689.2 ± 23.54	0.769927186	0.012
Proprotein Convertase 9	2763.8 ± 124.2	1747.45 ± 19.56	−0.661402044	0.037
FGF-21	404.1 ± 7.9	902.65 ± 45.1	1.159454344	0.036
IL-6	570.1 ± 33.81	272.55 ± 55.34	−1.064694081	0.048
IL-7	327.25 ± 23.5	661.2 ± 7.99	1.014693532	0.013
IL-10	553 ± 69.64	431.95 ± 54.31	−0.356415156	0.013
IL-11	674.45 ± 84.33	564 ± 8.6	−0.258016331	0.022
IL-15	270.95 ± 23.8	418.45 ± 31.5	0.6270286	0.013
IL-33	549.6 ± 55.7	299.5 ± 6.44	−0.875826001	0.030
IL-1ra/IL-1F3	448.85 ± 69.6	349.95 ± 23.7	−0.359084587	0.023
BAFF/BLyS/TNFSF13B	1639.55 ± 36.7	1067.55 ± 129.3	−0.618996257	0.027
Pentraxin 3/TSG-14	686.1 ± 55.9	1273.3 ± 24.59	0.892081599	0.028
Lipocalin-2/NGAL	1312.9 ± 36.8	2385.95 ± 59.7	0.861806776	0.001
Chitinase 3-like 1	1896.8 ± 45.32	723.2 ± 63.2	−1.391100985	0.032
Osteopontin (OPN)	3333.05 ± 93.5	990.35 ± 44.6	−1.750832576	0.043
CCL3/CCL4/MIP-1α/β	347.95 ± 7.64	194.6 ± 5. 46	−0.838368297	0.024
CXCL10/IP-10	554.7 ± 46.3	328.8 ± 32.57	−0.754497426	0.033
VEGF	549.35 ± 34.57	468.75 ± 93.4	−0.228906917	0.012
FGF acidic	491.39 ± 58.46	386.45 ± 8.55	−0.345441258	0.008
Amphiregulin	771.95 ± 5.89	639.55 ± 41.42	−0.271450252	0.013
IGFBP-5	2230.6 ± 101.3	3427.7 ± 145.3	0.61980902	0.013
VCAM-1/CD106	2762.8 ± 79.35	1307.4 ± 88.25	−1.079430526	0.000
Coagulation Factor III/	493.2 ± 13.56	412.1 ± 13.64	−0.259178336	0.032

All values are presented as mean ± standard deviation (SD).

Fat/Der-P: High-fat diet with Der p sensitization group.

FIP-fve + Fat/Der-P: FIP-fve-treated, high-fat diet with Der p sensitization group.

Statistical significance: *P* < 0.05 is considered significant.

### Histological results of airway inflammation in each mouse group

**Fig. 7:**
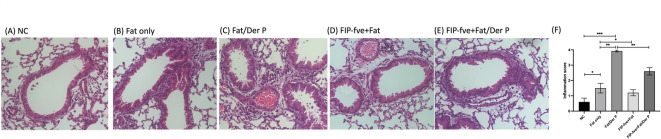
Histological Results of Airway Inflammation in Each Mouse Group. (**A**–**E**) representative H&E-stained lung sections from each group show: NC: Minimal inflammation and intact lung structure. Fat only: Mild inflammatory cell infiltration. Fat/Der P: Marked inflammation and airway thickening. FIP-fve + Fat: Inflammatory cell numbers reduced compared to Fat only. FIP-fve + Fat/Der P: Inflammation markedly reduced compared to Fat/Der P. (**F**) Inflammation scores quantified for all groups confirm these findings: the Fat/Der P group had the highest score, while both FIP-fve–treated groups showed significant reductions (*p < 0.05, **p < 0.01).

Figure 7 shows representative H&E-stained lung sections and inflammation scores across five groups. The NC group (A) displayed clear alveolar architecture with minimal inflammatory cell infiltration. The Fat only group (B) showed mild peribronchial and perivascular inflammation. In the Fat/Der P group (C), severe airway inflammation was observed, with dense inflammatory cell infiltration and marked tissue thickening. FIP-fve treatment notably reduced inflammation: FIP-fve + Fat (D) had decreased inflammatory cells and preserved alveolar structure, while FIP-fve + Fat/Der P (E) showed significantly less inflammation than the Fat/Der P group. Quantitative scoring (F) confirmed these histological findings: Fat/Der P mice had the highest inflammation scores, while both FIP-fve–treated groups exhibited significant reductions. These results indicate that FIP-fve effectively alleviates airway inflammation induced by combined obesity and allergen challenge.

## Discussion

Asthma is a prevalent and increasingly common chronic respiratory disease. Obesity significantly raises both the risk and severity of asthma—epidemiological studies show that abdominally obese children have a 1.6-fold higher risk of asthma^14^, and rapid BMI gain in early life similarly increases allergic asthma risk^15^. Mechanistically, obesity promotes airway narrowing due to decreased lung volume, induces low-grade chronic pulmonary inflammation, and alters adipokine profiles (such as increased leptin), all of which contribute to asthma development and exacerbation. Additionally, obesity-associated conditions (e.g., dyslipidemia, GERD, sleep-disordered breathing, and hypertension) can further worsen asthma control^1^. Animal models corroborate these findings: obese mice display increased airway hyperresponsiveness and heightened allergic responses, including delayed Th2 cytokine production, augmented neutrophil infiltration, and elevated IL-17 A at later stages—thereby intensifying allergic airway inflammation^9,16–21^. Obesity-driven upregulation of cytokines like IL-25 and TSLP in airway epithelium has also been implicated in asthma onset and progression^20,21^. These observations substantiate the intricate and synergistic relationship between obesity and allergic asthma.

This study provides compelling evidence of the synergistic interaction between obesity and allergic asthma, demonstrating that a high-fat diet exacerbates airway hyperresponsiveness (AHR) in mice, particularly when combined with Der p sensitization. This aligns with previous studies^9,14–21^ and epidemiological observations linking obesity to increased asthma prevalence and severity^7,22^.

This study utilized an obesity and allergic asthma combined model to comprehensively explore, for the first time, the regulatory role of Flammulina velutipes immunomodulatory protein (FIP-fve) in metabolic regulation, inflammatory responses, angiogenesis, tissue remodeling, and repair processes.

Our results clearly show that a high-fat diet combined with allergen exposure significantly increases airway and systemic inflammation, as well as metabolic abnormalities in mice. HFD led to greater body weight, serum cholesterol, glucose, and elevated levels of pro-inflammatory cytokines and adipokines, such as leptin, resistin, PCSK9, and DPPIV/CD26, consistent with findings that obesity drives chronic inflammation and metabolic dysregulation^1,8^. These effects were further intensified with Der p sensitization, underlining the mutually reinforcing pathology of obesity and allergy.

Importantly, FIP-fve intervention attenuated these pathological changes. FIP-fve reduced body weight, blood lipids, glucose, and crucial cytokines, including leptin and resistin, which are central to obesity-related insulin resistance and metabolic diseases. FIP-fve also suppressed PCSK9, a key factor in cholesterol metabolism and cardiovascular risk^2,6^, suggesting potential to lower cholesterol and cardiovascular risk in HFD-fed mice. The reduction of these markers paralleled significant drops in cholesterol, highlighting FIP-fve’s clinical potential in metabolic regulation.

Cytokine analysis revealed that, beyond metabolic benefits, FIP-fve also suppressed angiogenesis-related factors (Angiopoietin-1, Angiopoietin-2, VEGF, Periostin), which are associated with airway remodeling and inflammation^1,23^. Furthermore, FIP-fve lowered tissue repair and fibrosis markers such as Serpin E1/PAI-1, Osteopontin, and Chitinase 3-like-1—cytokines closely linked to chronic tissue damage and fibrosis^24–26^.

Although lipocalin-2, pentraxin-3, and osteoprotegerin are generally considered pro-inflammatory or obesity-related, their elevation in the FIP + Fat group (Fig. 5) may reflect regulatory or tissue-protective roles in certain settings^27,28^.

In summary, the multifunctional properties of FIP-fve observed in this study are consistent with its proposed role as a broad-spectrum immunometabolic modulator. By interacting with pattern recognition receptors such as TLR4 and Dectin-1, FIP-fve appears to promote anti-inflammatory immune phenotypes, including M2 macrophage polarization, which is associated with tissue repair and immune resolution. The suppression of major inflammatory pathways, specifically NF-κB and MAPK signaling, aligns with the observed reductions in pro-inflammatory cytokines such as IL-6, TNF-α, and IL-33, further supporting its anti-inflammatory capacity^12–15^. In parallel, FIP-fve influenced metabolic parameters by downregulating adipokines like leptin and resistin, as well as vascular and endothelial markers including VCAM-1, VEGF, PCSK9, and osteopontin, suggesting potential benefits in conditions characterized by metabolic dysregulation and vascular remodeling. Taken together, these mechanisms support the therapeutic potential of FIP-fve in modulating immune-metabolic interactions and maintaining tissue homeostasis in inflammatory disease states.

These findings underscore FIP-fve’s promise as a broad immunometabolic modulator with the unique ability to address both the inflammatory and metabolic aspects of obesity-related asthma. Compared with current therapies—such as corticosteroids (less effective in obesity), biologics (costly and do not address metabolic dysfunction), and statins (no asthma benefit)—FIP-fve combines airway and metabolic improvements with oral dosing convenience.

Further studies are warranted to validate its long-term safety and clinical relevance, but FIP-fve may offer a promising dual-action approach for managing obesity-related asthma, with benefits not fully addressed by existing therapies.

## Materials and methods

### Ethics statement

All animal experiments, including care and housing, were conducted in accordance with protocols approved by the Institutional Animal Care and Use Committee (IACUC) of Chung Shan Medical University (approval numbers 2127 and 2298). All procedures complied with institutional guidelines and the ARRIVE guidelines for reporting animal research to ensure ethical standards and animal welfare.

### Blinding or randomization

Animals were randomly assigned to experimental groups prior to treatment using a laboratory randomization protocol. Furthermore, experimental procedures, data analysis, and histopathological evaluations were performed in a blinded and randomized manner to minimize bias.

### Experimental animals

Female BALB/c mice (weighing approximately 20–25 g, aged 6–8 weeks) were purchased from the National Laboratory Animal Center, National Applied Research Laboratories, Taiwan. The mice were housed at the Animal Experiment Center of Chung Shan Medical University under controlled conditions: temperature of 22–24 °C, humidity of 55–60%, and a 12/12-hour light-dark cycle. Throughout the experiment, the mice were provided with sufficient food and water, and their bedding was changed regularly.

### Euthanasia and anesthesia use

#### Anesthesia

Mice were anesthetized using Thiopental (3.0–5.0 mg/100 g body weight, intraperitoneal injection) prior to any invasive procedures.

#### Analgesia

post-procedural pain management was ensured by administering Ketoprofen (2–5 mg/kg, subcutaneous injection) as necessary to alleviate any observed pain or distress.

#### Euthanasia

upon completion of the experimental timeline, mice were euthanized humanely using carbon dioxide (CO₂) inhalation, following guidelines to minimize discomfort and ensure ethical animal welfare standards.

### Purification and Preparation of flammulina velutipes Immunomodulatory protein (FIP-fve)

Fresh Flammulina velutipes mushrooms (300 g) were washed and soaked in 1 L of 5% ice-cold acetic acid solution, ensuring complete immersion, and stored at 4 °C for at least 1 h. The following steps were performed under refrigeration:

The mushrooms were blended into a slurry using a Waring blender. The mixture was centrifuged at 8,000 rpm for 20 min at 4 °C using a Beckman refrigerated centrifuge (JA14 rotor). The supernatant was filtered through filter paper, and solid ammonium sulfate was gradually added to achieve 90% saturation. The mixture was centrifuged at 8,000 rpm for 20 min to collect the precipitate, which was then dissolved in a small amount of distilled water. The solution was transferred to a dialysis bag (pre-softened in distilled water and checked for leaks) and dialyzed against 10 mM Tris-HCl (pH 8.0) at 4 °C for two days, with the dialysis solution changed every 12 h. After dialysis, the protein solution was centrifuged at 12,000 rpm for 40 min to remove insoluble impurities. The supernatant was passed through a cation-exchange DE52 column pre-equilibrated with 10 mM Tris-HCl (pH 8.0) and sequentially washed with the same buffer. Proteins bound to the column were eluted using a 0–0.5 N NaCl gradient in 10 mM Tris-HCl (pH 8.0), with a flow rate of 1 mL/min. The eluate was collected using a Gilson fraction collector in 1.5 mL aliquots, and protein absorbance was measured at 280 nm. The purity of the obtained FIP-fve was confirmed by SDS-PAGE.

### Experimental groups

The experiment consisted of five groups, each containing five mice, with all experiments performed at least three times: Normal Control (NC group): Mice fed a standard diet, Fat only group: Mice fed a high-fat diet, Fat/Der p group: Mice fed a high-fat diet and sensitized with Dermatophagoides pteronyssinus (Der p), FIP-fve + Fat group: Mice fed a high-fat diet and treated with FIP-fve and FIP-fve + Fat/Der p group: Mice fed a high-fat diet, sensitized with Der p, and treated with FIP-fve.

### Sensitization procedure and diet

BALB/c mice were assigned to groups receiving either a normal diet or a high-fat diet (HFD; 58% of energy from fat) for the entire experimental period. Sensitization to Dermatophagoides pteronyssinus (Der p) was conducted on days 1–3 and day 14 via intraperitoneal (i.p.) injection of 200 µL per mouse. The injected solution consisted of normal saline containing 0.8 mg Al(OH)₃ (for controls) or 50 µg Der p protein in saline with 0.8 mg Al(OH)₃.

On day 14, two procedures were performed: the final i.p. sensitization was administered in the morning, followed by the initial intranasal (i.n.) challenge with either 50 µL of normal saline or 5% Der p solution per mouse in the afternoon. This timing provided adequate recovery between procedures, minimized animal stress, and followed established mouse models for allergic airway inflammation.

Subsequent i.n. allergen (or saline) instillations were carried out on days 17, 21, 24, and 27, resulting in a total of five airway challenges. Airway hyper-responsiveness (AHR) was assessed on day 28. Mice were humanely euthanized within 24 h of the final assessment for downstream tissue and serum analysis.

Throughout, the fat diet group continued on high-fat chow, while the normal control (NC) group remained on standard diet. This protocol ensured synchronized modeling of systemic sensitization and local airway challenge, supporting robust and reproducible establishment of allergic airway inflammation in both dietary backgrounds.

### Diet composition details

The high-fat diet (HFD) was obtained from Yungli Animal Feed Company (distributor of Purina TestDiet^®^, Taiwan) and formulated to provide 60% of total energy as fat (approximately 35% fat by weight), with the remaining calories from protein (16–20%) and carbohydrates (20–24%).

### FIP-fve administration

Based on the sensitization protocol, mice were orally administered FIP-fve via gavage at a dose of 200 µg per mouse (100 µL per dose) from Days 14–27. After 14 consecutive days of treatment, FIP-fve administration was stopped, while the sensitization process continued. On Day 28, AHR was measured, and mice were sacrificed for sample collection.

### Airway hyper-responsiveness (AHR) measurement

On Day 28, AHR was assessed by exposing mice to increasing concentrations of methacholine (0, 5, 10, and 20 mg/mL; Sigma-Aldrich, Inc.) via nebulized inhalation for 3 min. Mice were then placed in a vacuum chamber connected to an airway response detection system (BUXCO Electronics, Inc., Wilmington, NC, USA) for 3 min. Respiratory rate and airflow changes were recorded by sensors and analyzed using BioSystem XA software to determine AHR values (enhanced pause), which were used to calculate thoracic pressure changes during inhalation and exhalation induced by methacholine.

### Blood sample collection and analysis

On the day of AHR measurement, mice were sacrificed, and whole blood was collected via the inferior vena cava. Blood samples were centrifuged (3,000 rpm, 4 °C, 10 min) to isolate serum for further analysis. Serum concentrations of ALT, AST, TG, and cholesterol were measured at a veterinary hospital.

### Cytokines and array analysis

The Proteome Profiler Mouse XL Cytokine Array (ARY028; R&D Systems, USA) was used to detect cytokine expression levels in mouse serum. This array detects 111 cytokines and chemokines simultaneously, and all procedures were conducted according to the manufacturer’s instructions. In addition to the cytokine array, we also used R&D Systems ELISA kits to measure several cytokines, including IL-4, IL-5, IL-6, IL-13, IL-17, IL-33, and TNF-α. The assays were performed according to the manufacturer’s instructions.

### Histological assessment

For the histological assessment, lung tissues were harvested from each mouse immediately after euthanasia and fixed in 4% paraformaldehyde for at least 24 h to ensure optimal preservation of cellular morphology. After fixation, the tissues were processed and embedded in paraffin blocks. Serial sections with a thickness of 4–5 μm were cut using a microtome. These sections were then deparaffinized, rehydrated through a graded series of alcohols, and stained with Hematoxylin and Eosin (H&E) to clearly visualize general tissue architecture and the extent of inflammatory cell infiltration.

For evaluation, multiple airway cross-sections—specifically including peribronchial and perivascular areas—were examined from each animal. The degree of airway inflammation was scored semi-quantitatively using a standardized grading system commonly applied in murine asthma research: a score of 0 represented no inflammatory cells; 1 indicated a few inflammatory cells; 2 denoted a ring of inflammatory cells one cell layer deep; 3 indicated a ring two to four cells deep; and 4 referred to a ring of more than four cell layers deep or densely packed inflammatory infiltrates. All slides were independently scored by at least two blinded investigators to minimize subjective bias. The mean value from all examined cross-sections for each animal was calculated to yield a representative inflammation score for further statistical analysis.

### Statistical analysis

Data were analyzed using GraphPad Prism software (Boston, MA). Results are presented as median ± interquartile range (IQR). The Mann-Whitney test was used for statistical comparisons, with significance levels set at *p* ≤ 0.05 (*) and *p* ≤ 0.01 (**), indicating significant differences between groups.

## Data Availability

Data availability: the datasets used and/or analysed during the current study available from the corresponding author on reasonable request.
